# The Management of Intracardiac Thrombus in a COVID-19 Patient Using IV Thrombolytics: A Case Report

**DOI:** 10.7759/cureus.64085

**Published:** 2024-07-08

**Authors:** Vandana Bandari, Sai Rakshith Gaddameedi, Shaji Faisal, Ashmin Singh, Muhammed Z Ghatala, Manjeet Singh, Shazia M Shah

**Affiliations:** 1 Internal Medicine, Bayhealth Medical Center, Dover, USA; 2 Internal Medicine, Rutgers Health/Monmouth Medical Center, Long Branch, USA; 3 Internal Medicine, Gandhi Medical College, Secunderabad, IND; 4 Cardiology, Bayhealth Medical Center, Dover, USA

**Keywords:** covid-19, coronavirus, thrombolysis, thrombolytics, pulmonary embolism, cardiac thrombus, intracardiac thrombus

## Abstract

The coronavirus disease 2019 (COVID-19) pandemic has unveiled numerous clinical challenges, particularly its association with thrombotic events, which significantly contribute to morbidity and mortality. While thrombotic complications such as arterial and venous thromboembolism (VTE) are well-documented, instances of intracardiac thrombus are notably rare. This case report discusses a 60-year-old male with COVID-19 who came to the hospital due to respiratory distress. Despite treatment with remdesivir, the patient's condition worsened prompting further workup. His nuclear medicine (NM) ventilation-perfusion scan was inconclusive, but a 2D echocardiogram showed an intracardiac thrombus in the right atrium (RA) and right ventricle (RV). As the patient's condition worsened, necessitating a transition from nasal cannula to high-flow nasal cannula, a decision was made to treat him with intravenous (IV) thrombolytic therapy. The patient received 100 mg IV alteplase and IV heparin, resulting in significant respiratory improvement and symptomatic relief. A repeat echocardiogram after 48 hours showed normal ejection fraction and complete thrombus resolution. In conclusion, this case highlights the complex link between COVID-19 infection and prothrombotic states, leading to severe complications such as intracardiac thrombus in transit. The successful treatment of this patient through a multidisciplinary approach and thrombolytic therapy underscores the importance of prompt recognition and intervention in high-risk cases.

## Introduction

The emergence of coronavirus disease 2019 (COVID-19) has presented unprecedented challenges to healthcare systems worldwide, revealing a myriad of clinical manifestations extending beyond respiratory compromise. Among these complexities, mounting evidence indicates that thrombotic events have become significant contributors to morbidity and mortality. Numerous investigations have documented heightened procoagulant activity in COVID-19 patients, characterized by elevated plasma levels of soluble thrombomodulin, suggesting increased endothelial cell activation, fibrin production, and resistance to fibrinolysis [1].

While there have been numerous reports of COVID-19-related complications, such as arterial and venous thromboembolism (VTE) and cardiomyopathy, instances of intracardiac thrombus have been relatively rare. Herein, we present a case of a 60-year-old male with COVID-19 who developed a large intracardiac thrombus in transit, shedding light on the complexities of managing thrombotic complications in this viral illness. The abstract of this case was accepted and presented at the 2024 National American College of Physicians (ACP) Conference in Boston.

## Case presentation

A 60-year-old male patient with a medical history notable for hypertension and gastroesophageal reflux disease (GERD) presented with a constellation of symptoms persisting for two weeks. These symptoms included dyspnea, fever, chills, and fatigue. Upon initial assessment, the patient exhibited signs of respiratory distress, manifested by tachypnea and tachycardia. Oxygen saturation was 90% while receiving 4 L of oxygen through a nasal cannula.

Laboratory investigations unveiled several concerning findings: Creatinine levels were elevated at 1.4 mg/dL from a baseline of 0.7 mg/dL, calcium levels were elevated at 13 mg/dL, lactate dehydrogenase (LDH) was elevated at 248 IU/L, CRP was markedly elevated at 186 mg/L, D-dimer levels were significantly elevated at 13,593 ng/mL, pro-brain natriuretic peptide (BNP) levels were elevated at 4,064 pg/mL, troponin was mildly elevated at 0.17 ng/mL, and WBC count was elevated at 20,700 per microliter.

**Figure 1:**
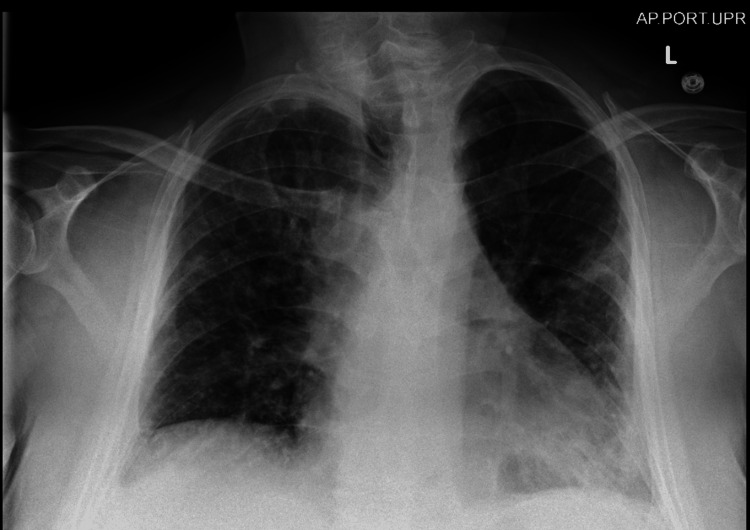
Chest X-ray The cardiomediastinal silhouette is enlarged. There are mild patchy infiltrates throughout the left lung, as well as in the right midlung field. The pleural spaces are clear. The bones and soft tissues are within normal limits

**Figure 2:**
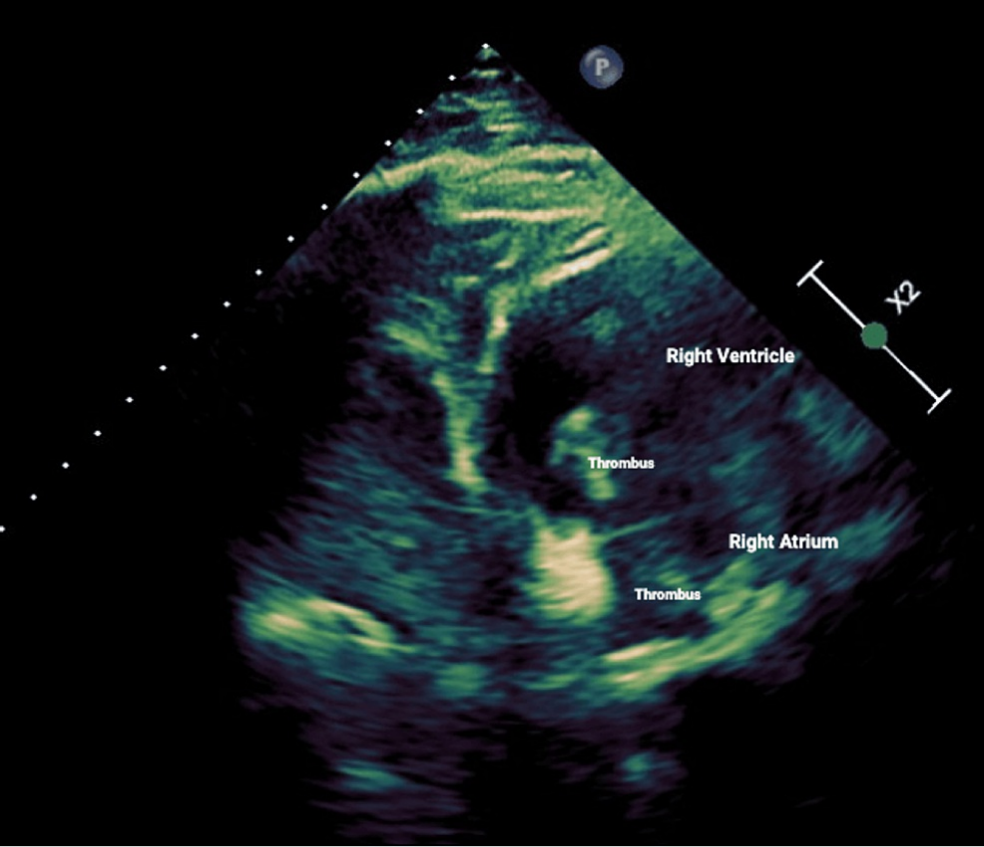
2D echocardiogram view showing intracardiac thrombus extending within the right atrium and right ventricle

Imaging studies, including a chest X-ray (Figure 1), depicted patchy infiltrates bilaterally, suggestive of pulmonary involvement. Computed tomography pulmonary angiogram (CTPA) was not performed as the patient had acute kidney injury with an elevation of creatinine from his baseline, so a nuclear medicine (NM) ventilation-perfusion scan was performed instead. The NM ventilation-perfusion scan yielded inconclusive results regarding pulmonary embolism (PE). Subsequent testing confirmed a positive diagnosis of COVID-19, prompting the initiation of isolation and treatment with intravenous (IV) steroids and remdesivir. Despite treatment, the patient's symptoms persisted, characterized by worsening shortness of breath and lightheadedness with minimal exertion. Further diagnostic evaluation via bilateral lower extremity Doppler ultrasound showed extensive deep venous thrombus within the right common femoral, popliteal, post-tibial, and peroneal veins. The patient was subsequently started on anticoagulation with therapeutic Lovenox. Notably, a transthoracic echocardiogram (Figure 2) revealed a normal ejection fraction, with a multilobar, mobile, solid thrombus in the right atrium (RA) and right ventricle (RV) measuring about 6.3×2.8 cm.

His condition deteriorated rapidly, requiring increased oxygenation and placement on a high-flow nasal cannula. Recognizing the severity of the patient's condition and the multisystem involvement, a multidisciplinary approach involving cardiothoracic surgery, pulmonology, and cardiology was brought in to help curate a treatment plan. After careful consideration, it was decided to pursue thrombolytic therapy using intravenous alteplase alongside therapeutic anticoagulation with intravenous heparin.

He was treated with IV thrombolytic therapy with 100 mg alteplase and IV heparin.

**Figure 3:**
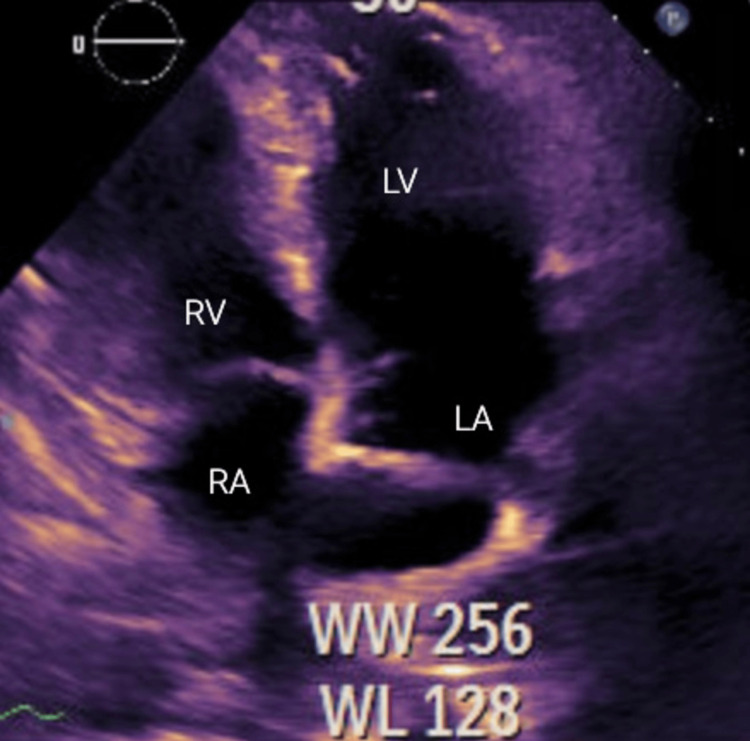
2D echocardiogram view post thrombolytic therapy showing the resolution of clot RA, right atrium; LA, left atrium; RV, right ventricle; LV, left ventricle

Following this intervention, the patient exhibited a notable improvement in respiratory status and reported symptomatic relief. He was weaned back to nasal cannula and eventually to room air. A repeat echocardiogram (Figure 3) 48 hours later revealed the left ventricular ejection fraction restoration within normal limits, with the complete resolution of intracardiac thrombus.

Throughout hospitalization, the patient's clinical trajectory demonstrated a favorable evolution, culminating in the resolution of symptoms and restoration to baseline functional status. Upon discharge, the patient was asymptomatic and prescribed a three-month course of oral anticoagulation for the management of provoked venous thrombosis.

## Discussion

Coronavirus disease 2019 (COVID-19), caused by the severe acute respiratory syndrome coronavirus 2 (SARS-CoV-2), is now understood as a systemic disorder that induces a prothrombotic state, leading to increased mortality. Various studies have reported heightened procoagulant activity in COVID-19 patients, characterized by elevated plasma levels of soluble thrombomodulin, indicative of enhanced endothelial cell activation, augmented fibrin formation, and increased resistance to fibrinolysis. These factors collectively contribute to the development of COVID-19-associated coagulopathy. Clinical manifestations of COVID-19 coagulopathy encompass arterial and venous thrombotic events [2]. According to a single-center study conducted on 388 COVID-19 patients by Lodigiani and colleagues (2020), it was observed that despite the administration of anticoagulant prophylaxis, a considerable incidence of thromboembolic events, primarily venous thromboembolism (VTE), has been reported within 24 hours of admission [3].

Autopsy analysis of individuals who succumbed to COVID-19 revealed a significant occurrence of deep venous thrombosis (58%) and fatal pulmonary embolism (33%) [4]. Likewise, a separate investigation revealed a heightened prevalence of pulmonary embolism (20.4%) among COVID-19 patients admitted to intensive care units. There have already been cases in the literature describing transient right atrial thrombus in patients with COVID-19 infection or mobile right ventricular thrombus causing a pulmonary embolism [5].

Given that right ventricular (RV) thrombi often originate in the deep venous system and transit peripherally, they are commonly referred to as "in transit" [6]. Echocardiography has proven invaluable in diagnosing and managing patients with pulmonary embolism (PE) and hemodynamic instability. A dilated right ventricle (RV), McConnell's sign, and a thrombus can exhibit sensitivities of 80%, 97%, and 99%, respectively, along with positive likelihood ratios of 4, 7.3, and 5 [7]. A right ventricular thrombus in transit, while rare, occurs in 2%-4% of PE patients, particularly those with hypotension or RV dysfunction (20%). Its presence not only confirms the diagnosis of PE but also carries a poor prognosis. Notably, cases of thrombus in transit have been documented in patients with COVID-19. Point-of-care ultrasound (POCUS) has emerged as a valuable tool in evaluating patients presenting with shock and suspected PE. It accurately detects most signs of RV pressure overload, including RV dilatation, D shape, McConnell's sign, thrombus in transit, and inferior vena cava dilatation. Its portability and rapid acquisition protocols shorten the time from presentation to diagnosis and treatment, ultimately improving patient outcomes [8]. A retrospective analysis of 177 cases of RV thrombi revealed that PE was present in 98% of cases. Treatment decisions for right heart thrombus in COVID-19 patients are based on individual cases, considering the risks and benefits of surgery versus thrombolysis. In patients without contraindications to thrombolytic agents, a trial of thrombolysis may precede surgery, with improved survival rates observed among those treated with thrombolysis compared to anticoagulation and thrombectomy alone [9].

Assessing the risk of death from pulmonary embolism (PE) requires a comprehensive evaluation of various contributing factors. While refractory hypotension and shock in patients with low bleeding risk may indicate a need for thrombolytic therapy, none of the individual factors listed alone are a definitive indication. Instead, a holistic approach, considering all factors collectively and accurately to assess the risk of death from PE, is essential. These factors include persistent hypotension or shock despite resuscitation, new or worsening right ventricular (RV) dysfunction detected through various diagnostic methods, elevated biomarkers such as troponin and BNP, a high simplified Pulmonary Embolism Severity Index (sPESI) score, a high clot burden, proximal deep venous thrombosis, significant hypoxemia, tachycardia, poor cardiopulmonary reserve, and the presence of a right-sided cardiac thrombus. By considering these factors collectively, clinicians can make informed decisions regarding the appropriate management strategies for PE patients and aim to reduce mortality risk [10].

When considering patients for thrombolysis, expert clinicians rely on a combination of clinical variables and an overall assessment to stratify patients based on their risk of death from PE. Those categorized as high risk, indicating hemodynamic instability, are considered suitable candidates for thrombolytic therapy if the bleeding risk is low. In the intermediate-risk category, the benefit derived from thrombolysis varies significantly, necessitating additional assessment to identify those most likely to benefit. The intermediate-risk group is subdivided into intermediate-high and intermediate-low risk. Intermediate-high risk includes patients with abnormal RV function and elevated brain natriuretic peptide (BNP) or troponin [10]. In contrast, intermediate-low-risk patients have abnormal RV function or elevated troponin or BNP. The intermediate-high-risk group potentially benefits from thrombolysis. Those classified as low risk typically do not require thrombolysis and are managed with anticoagulation alone [11].

In our case, the patient stratified as an intermediate-high risk with refractory hypoxemia and no contraindications to thrombolytic therapy. He experienced an improvement in symptoms and a resolution of the thrombus following thrombolytics, as confirmed on repeat echocardiogram. Right heart thrombi in transit in PE patients identifies a subgroup with markedly high short-term mortality. The timely identification of venous thromboembolic events causing resistant hypoxia is crucial for initiating prompt thrombolytic therapy. Given the lack of established guidelines for systemic thrombolysis in cases of thrombus in transit as a complication of COVID-19, further research is warranted to assess its efficacy in such scenarios.

## Conclusions

In conclusion, this case underscores the intricate association between COVID-19 infection and prothrombotic states, resulting in severe complications such as intracardiac thrombus in transit. The successful management of this patient, utilizing a multidisciplinary approach and thrombolytic therapy, highlights the importance of prompt recognition and intervention in high-risk cases. Despite the lack of established guidelines for systemic thrombolysis for thrombus in transit associated with COVID-19, this case suggests that such interventions can lead to favorable outcomes in carefully selected patients. Further research is warranted to delineate better the optimal management strategies for this complex clinical scenario.
